# Reference gene stability of a synanthropic fly, *Chrysomya megacephala*

**DOI:** 10.1186/s13071-015-1175-9

**Published:** 2015-10-29

**Authors:** Xiaoyun Wang, Mei Xiong, Jialu Wang, Chaoliang Lei, Fen Zhu

**Affiliations:** Hubei Insect Resources Utilization and Sustainable Pest Management Key Laboratory, Huazhong Agricultural University, Wuhan, 430070 China

**Keywords:** *Chrysomya megacephala*, Synanthropic, Reference genes, qPCR

## Abstract

**Background:**

Stable reference genes are essential for accurate normalization in gene expression studies with reverse transcription quantitative polymerase chain reaction (qPCR). A synanthropic fly, *Chrysomya megacephala*, is a well known medical vector and forensic indicator. Unfortunately, previous studies did not look at the stability of reference genes used in *C. megacephala*.

**Results:**

In this study, the expression level of Actin, ribosomal protein L8 (Rpl8), glyceraldehyde-3-phosphate dehydrogenase (GAPDH), elongation factor 1α (EF1), α-tubulin (α-TUB), β-tubulin (β-TUB), TATA binding box (TBP), 18S rRNA (18S) and ribosomal protein S7 (Rps7) were evaluated for their stability using online software RefFinder, which combines the normal software of the ΔCt method, BestKeeper, Normfinder, and geNorm. Moreover the number of suitable reference gene pairs was also suggested by Excel-based geNorm. The expression levels of these reference genes were evaluated under different experimental conditions with special perspectives of forensic applications: developmental stages (eggs, first, second and third instar larvae, pupae and adults); food sources of larvae (pork, fish and chicken); feeding larvae with drugs (untreated control, Estazolam and Marvelon); feeding larvae with heavy metals (untreated control, cadmium and zinc); tissues of adults (head, thorax, abdomen, legs and wings). According to RefFinder, EF1 was the most suitable reference gene of developmental stages, food and tissues; 18S and GAPDH were the most suitable reference genes for drugs and heavy metals, respectively, which could be widely used for quantification of target gene expression with qPCR in *C. megacephala*. Suitable reference gene pairs were also suggested by geNorm.

**Conclusion:**

This fundamental but vital work should facilitate the gene studies of related biological processes and deepen the understanding in physiology, toxicology, and especially medical and forensic entomology of *C. megacephala*.

**Electronic supplementary material:**

The online version of this article (doi:10.1186/s13071-015-1175-9) contains supplementary material, which is available to authorized users.

## Background

The oriental latrine fly, *Chrysomya megacephala* (Calliphoridae) is of medical importance and distributed worldwide [1]. *C. megacephala* feed on and breed in filth, *ie*, carcasses and decaying organic matter, animal manure and garbage, which are full of pathogens [2]. Thus they mechanically transmit human and animal pathogens *ie*, viruses, bacteria, protozoan cyst and helminth eggs, by adhering them on their surfaces or ingesting them into their guts, and then cause public health issues and economic loss [2–6]. For instance, *C. megacephala* could contaminate food with *Toxoplasma. gondii* oocysts by contacting with infectious fecal material, transmitting disease across animal species [7] *C. megacephala* could also cause human and animal myiasis based on their breeding characteristic. The first case of the human aural myiasis was from a teenage girl infested by third instar larvae of *C. megacephala* in Malaysia [8]. Some other myiasis cases were reported later in more areas of the world with *C. megacephala* and other *Chrysomya* species [9–13].

*Chrysomya megacephala* is also a forensically-important fly species because of its breeding characteristic. They are first in the queue to locate a human corpse, making them valuable during the estimate postmortem interval (PMI) [14]. Among all the aspects of forensic entomology, the growth and development of the larvae is of great interest, especially for PMI of human cadavers [14]. Biotic and abiotic factors affect the PMI determination [15]. Abiotic factors, such as environmental conditions and toxins or drugs in the corpse might diverge from the empirical situation, leading to errors in the PMI estimate [16]. The effects of drugs, heavy metals and natural products on the development of *Chrysomya* species have been observed to avoid the potential deviation and achieve a more accurate PMI estimate [15, 17–19].

Transcriptomes of *C. megacephala* provided a great quantity of sequence information [20, 21]. The developmental gene *bicoid* has been used to identify species of forensically important blowflies, avoiding confusion in morphological parameters (Diptera: calliphoridae) [22]. Molecular biological technique and methods would contribute to investigate the related mechanisms in both medical and forensic applications of *C. megacephala*. However, reference gene stability in *C. megacephala* under fundamental conditions has not been evaluated yet.

Generally, quantitative real-time reverse transcriptase PCR (qPCR) is an accurate measurement in small changes of mRNA levels [15, 16]. Moreover, normalization is the key to achieve an accurate result of target gene with stable standard gene [23]. Traditionally, housekeeping genes have been normally used as reference genes for the normalization of qPCR data, for example*,* actin, ribosomal proteins, glyceraldehyde-3-phosphate dehydrogenase (GAPDH), elongation factor1α (EF1), α-tubulin (α-TUB), β-tubulin (β-TUB), TATA binding box (TBP) and 18SrRNA (18S) [24]. However, it is possible for the expression of these reference genes to vary in different environmental conditions [25].

In this study, we aimed to screen stable reference genes in *C. megacephala*. Nine candidate genes (Actin, Rpl8, GAPDH, EF1, α-TUB, β-TUB, TBP, Rps7 and 18S) were evaluated under five laboratory conditions: developmental stages (eggs, first, second and third instar larvae, pupae and adults); food sources of larvae (pork, fish and chicken); feeding larvae with drugs (untreated control, Estazolam and Marvelon); feeding larvae with heavy metals (untreated control, cadmium and zinc); and tissues of adults (head, thorax, abdomen, legs and wings). One freely available online tool, RefFinder, which combines geNorm [26], NormFinder [27], and BestKeeper [28], and the ΔCt method [29] and Excel-based geNorm was used to evaluate the number of suitable reference gene pairs for gene expression studies in *C. megacephala*.

## Results

### Transcriptional levels of candidate reference genes

**Fig. 1:**
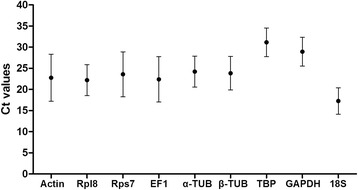
Expression levels of candidate reference genes in total samples of *C. megacephala.* Expression levels of the candidate *C. megacephala* reference genes of all samples are displayed as cycle threshold (Ct) values (Mean ± SD). The black dot indicates the mean Ct values for each candidate gene, and the bars indicate the SD

The primers of the nine candidate reference genes were tested to be suitable for gene stability assay with ideal amplification parameters (Table 1). Transcriptional levels for the nine candidate reference genes were showed within all samples of *C. megacephala* (Fig. 1). Among them, 18S (17.26) and Rpl8 (21.18) were the most and second most highly expressed; and TBP (31.15) and Rps7 (28.93) were the lowest and penultimate lowest expressed. 18S and TBP showed the most narrow expression variability within all samples; while Actin and EF1 showed the most broad expression variability within all samples.

**Table 1:** Primers of the candidate reference genes for qPCR

Gene	Accession number	Primer sequences (5' → 3')	PCR products (bp)^a^	E^a^ (%)	R^2 b^
*Actin*	KC207081	F: ACACCATCACCAGAATCCAAG	149	97.6	0.994
		R: TTAAACCCCAAGGCTAACCG			
*Rpl8*	KM289151	F: CTCCAAATCGGCAATGTGATG	148	93.9	0.992
		R: TCTTGGTGTCAGGGTTGTG			
*GAPDH*	KM289150	F: AGTTATCCCTGCCTTGAACG	132	97.1	0.998
		R: AAGACCTTAGCCTTGATGTCG			
*EF1*	FR719225	F: TTCACCGCTCAAGTCATCG	122	95.0	0.998
		R: TCGACCTTCTCCTTGATTTCAG			
*α-TUB*	KM289152	F: GAAGGTGAATTCTCTGAGGCC	144	98.0	0.974
		R: GTCTTTTGGTTTGTGGAACGAG			
*β-TUB*	KM289153	F: CCATTTCATCCATACCCTCACC	141	92.5	0.994
		R: GCTTGAAAATGTCTGCCACC			
*TBP*	KM387673	F: TCATCCGCAACTCCATCTTC	127	98.7	0.991
		R: TGGGCGACATAAGACTTTGTG			
*18S*	FJ025483	F: AGCGTATTACCGGTGGAGTTCT	78	92.5	0.994
		R:CTGAAGCAGGTTTAAATAGGAGGA			
*Rps7*	KM289154	F: CCTTTTCACGAGCCGCTTCC	76	90.4	0.999
		R: GTGCCGGTACCCGTTACTGA			

^a^qPCR efficiency (calculated by the standard curve method)

^b^Regression coefficient of theqPCR reaction

### Stability of reference genes

#### Developmental stages

**Fig. 2:**
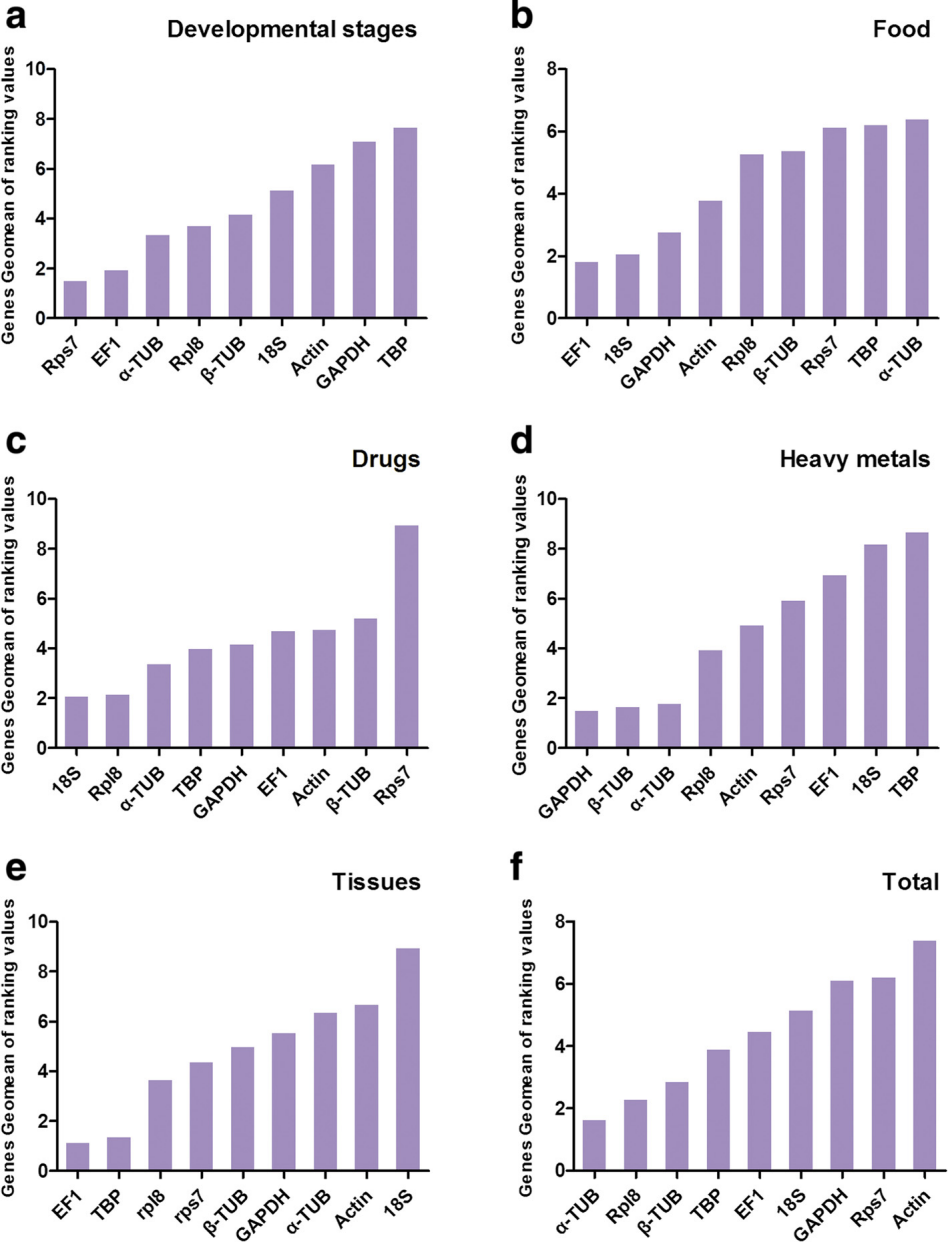
Expression stability ranking orders of the candidate reference genes calculated by the Geomean method of RefFinder. A lower Geomean ranking indicates more stable expression. Expression stability of reference genes were listed in the following samples: **a**-developmental stages of *C. megacephala*; **b**-*C.megacephala* treated with different food resources; **c**-*C. megacephala* treated with different drugs; **d**-*C. megacephala* treated with heavy metals; **e**-tissues of adult *C. megacephala*; **f**-total samples

**Fig. 3:**
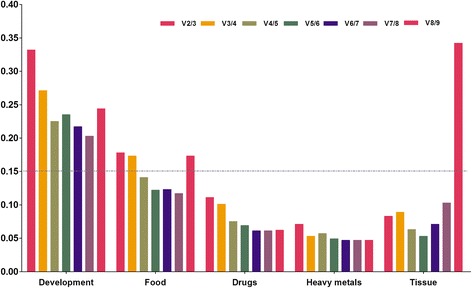
The optimal number of reference genes for normalization by geNorm analysis. Average pairwise variations (V) were calculated by geNorm between the normalization factors NF_n_ and NF_n+1_ to indicate whether inclusion of an extra reference gene would add to the stability of the normalization factor. Values < 0.15 indicate that additional genes are not required for the normalization of gene expression

Rps7 and EF1 were the most stable reference genes of developmental stages according to the comprehensive ranking orders from RefFinder, (Fig. 2a). For separate software analysis, the ΔCt method, NormFinder and geNorm also suggested Rps7 and EF1 as the most suitable ones; while Bsetkeeper suggested 18S and Rpl8 as the most suitable ones (Table 2). geNorm would also give the optimal numbers of control genes for normalization. geNorm manual suggested that if V_n/n+1_ < 0.15, it should be unnecessary to use ≥ n + 1 genes as control genes. Therefore, reference genes were expressed unstably in *C. megacephala*. We would like to suggest the three top-ranking ones to be used as internal controls in further studies based on the moneywise principle (Fig. 3).

**Table 2:** Ranking orders of the reference genes of *C. megacephala* in different experimental conditions

Conditions		ΔCt		Bestkeeper		NormFinder		geNorm	
	Rank	Gene	Stability	Gene	Stability	Gene	Stability	Gene	Stability
Developmental	1	EF1	2.597	18S	1.758	Rps7	1.384	EF1|	1.550
								Rps7	
Stages	2	Rps7	2.650	Rpl8	2.351	EF1	1.392		
	3	α-TUB	2.702	Rps7	3.169	α-TUB	1.455	α-TUB	1.655
	4	β-TUB	2.840	β-TUB	3.191	β-TUB	1.532	Rpl8	1.975
	5	Rpl8	2.849	α-TUB	3.327	Rpl8	1.592	β-TUB	2.179
	6	Actin	3.266	Actin	3.395	Actin	2.357	GAPDH	2.406
	7	GAPDH	3.296	TBP	3.899	GAPDH	2.575	Actin	2.549
	8	TBP	3.383	EF1	3.904	TBP	2.645	TBP	2.680
	9	18S	4.828	GAPDH	4.664	18S	4.483	18S	3.157
Food	1	EF1	1.941	18S	0.449	EF1	0.874	Actin|	0.766
								GAPDH	
Resources	2	18S	2.122	Rps7	1.090	18S	1.118		
	3	GAPDH	2.127	TBP	1.582	GAPDH	1.512	EF1	1.060
	4	Actin	2.260	EF1	1.687	β-TUB	1.548	Rpl8	1.221
	5	Rpl8	2.276	α-TUB	1.766	Rpl8	1.639	18S	1.603
	6	β-TUB	2.307	β-TUB	1.860	Actin	1.730	β-TUB	1.796
	7	α-TUB	2.570	GAPDH	2.077	α-TUB	1.983	α-TUB	2.025
	8	TBP	2.685	Rpl8	2.090	TBP	2.139	TBP	2.182
	9	Rps7	3.016	Actin	2.338	Rps7	2.602	Rps7	2.367
Different	1	Rpl8	1.194	18S	0.477	18S	0.659	Rpl8|	0.567
								EF1	
Drugs	2	α-TUB	1.200	β-TUB	0.637	α-TUB	0.668		
	3	18S	1.238	TBP	0.839	TBP	0.758	Actin	0.705
	4	GAPDH	1.239	GAPDH	0.948	Rpl8	0.785	GAPDH	0.789
	5	TBP	1.246	Actin	0.980	GAPDH	0.798	α-TUB	0.977
	6	Actin	1.295	Rpl8	1.057	Actin	0.918	TBP	1.054
	7	β-TUB	1.483	α-TUB	1.071	β-TUB	1.178	18S	1.113
	8	EF1	1.507	EF1	1.094	EF1	1.314	β-TUB	1.229
	9	Rps7	1.800	Rps7	1.184	Rps7	1.646	Rps7	1.356
Different	1	GAPDH	0.604	β-TUB	0.233	GAPDH	0.212	α-TUB|	0.260
								β-TUB	
Heavy metals	2	α-TUB	0.611	GAPDH	0.269	α-TUB	0.297		
	3	β-TUB	0.636	α-TUB	0.338	β-TUB	0.351	GAPDH	0.336
	4	Rpl8	0.691	Rpl8	0.408	Rpl8	0.423	Rpl8	0.411
	5	Actin	0.728	Actin	0.462	Actin	0.476	Actin	0.491
	6	Rps7	0.744	Rps7	0.528	Rps7	0.556	Rps7	0.577
	7	EF1	0.790	EF1	0.587	EF1	0.607	EF1	0.618
	8	18S	0.841	TBP	0.613	18S	0.705	18S	0.650
	9	TBP	1.095	18S	0.655	TBP	1.015	TBP	0.749
Different	1	TBP	1.232	EF1	0.523	EF1	0.059	EF1|	0.272
								TBP	
Tissues	2	EF1	1.269	TBP	0.558	TBP	0.136		
	3	Rpl8	1.306	α-TUB	0.634	Rpl8	0.221	Rpl8	0.428
	4	Rps7	1.412	Actin	0.749	Rps7	0.401	Rps7	0.639
	5	GAPDH	1.532	β-TUB	0.760	GAPDH	0.715	GAPDH	0.787
	6	α-TUB	1.572	Rps7	0.863	β-TUB	1.190	β-TUB	0.932
	7	β-TUB	1.753	Rpl8	0.887	α-TUB	1.477	α-TUB	1.026
	8	Actin	2.001	GAPDH	0.957	Actin	1.590	Actin	1.193
	9	18S	3.729	18S	3.714	18S	3.655	18S	1.756

#### Food resource

EF1 and 18S were the most stable reference genes of food resource according to the comprehensive ranking orders from RefFinder (Fig. 2b). For separate software analysis, the ΔCt method and NormFinder suggested the same reference gene pair as the most suitable while Bestkeeper suggested 18S and Rps7, geNorm suggested Actin and GAPDH as the most stable pairwise (Table 2). According to geNorm, five reference genes should be required for normalization of food resource treatment. However, we would like to suggest the three top-ranking ones based on the moneywise principle (Fig. 3).

#### Drugs

18S and Rpl8 were the most stable reference genes of drug treatment according to the comprehensive ranking orders from RefFinder (Fig. 2c). For separate software analysis, the ΔCt method suggested that Rpl8 and α-TUB were the most stable; Bestkeeper suggested 18S and β-TUB; NormFinder suggested 18S and α-TUB; geNorm suggested Rpl8 and EF1. According to geNorm, the two top-ranking ones should be required for normalization in drug-stressed larval samples (Fig. 3).

#### Heavy metals

GAPDH and β-TUB were the most stable reference genes of heavy metal treatment (Fig. 2d) according to the comprehensive ranking orders from RefFinder. For separate software analysis, Bestkeeper suggested the same genes; while the ΔCt method and NormFinder suggested GAPDH and α-TUB; geNorm suggested α-TUB and β-TUB (Table 2). According to geNorm, the two top-ranking ones should be required for normalization in heavy metal-stressed larval samples.

#### Tissues

According to the comprehensive results of RefFinder, EF1 and TBP were the most stable reference genes of adult tissues (Fig. 2e). For separate software analysis, all of them suggested that EF1 and TBP were the most stable reference genes (Table 2). According to geNorm, the two top-ranking ones should be required for normalization in heavy metal-stressed larval samples.

#### Total

According to the comprehensive results of RefFinder, α-TUB and Rpl8 were the most stable reference genes of total samples (Fig. 2f). However, they might not be directly used in separate experiments even though they were suggested because their performance as reference genes varied in different conditions. It might be a shortcut for a likely audience to verify whether these two genes would be appropriate for their experiments without all the reference gene evaluation in large scales as we did.

Ranking orders of all larval, pupal and adult samples by RefFinder were listed in the Additional files 1, 2 and 3, respectively. According to the results, the two most stable reference genes of total samples larval, pupal and adult were Rps7 and β-TUB; Rpl8 and EF1; EF1 and Rpl8; respectively. Similarly, verify them beforehand.

### Reference gene validation

**Fig. 4:**
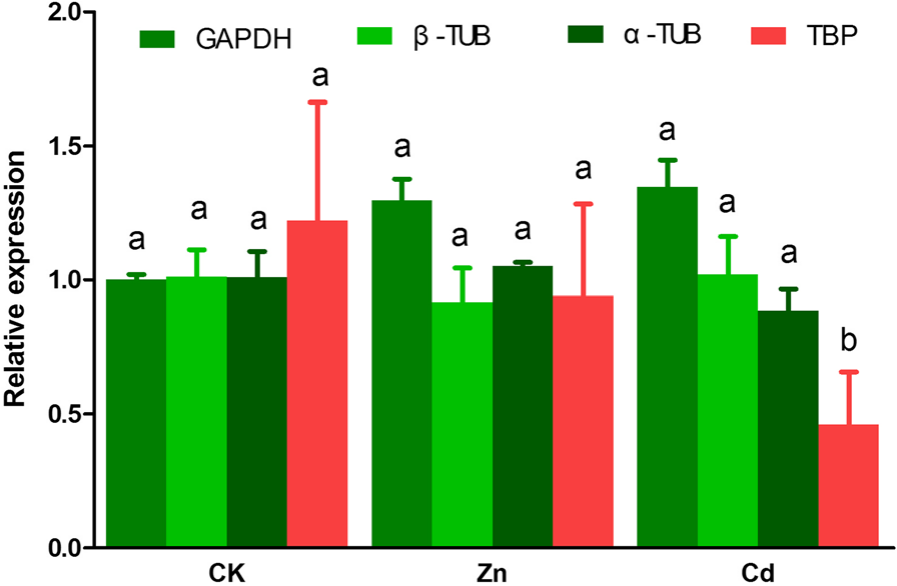
The expression level of *Hsp70* by normalization with four reference genes. Data represent mean values ± SEM calculated from three biological replicates. Those in the columns followed by the different letters indicate significant difference (*p* = 0.05, Duncan’s multiple range test)

Most of the reference selection studies would randomly choose a target for stability validation [30, 31]. Here, heat shock protein 70 (Hsp70) was selected to evaluate the reliability of the identified reference genes (GAPDH, β-TUB and α-TUB). Heat shock proteins are highly conserved and might be induced by stress in organisms [32]. However, *Hsp* also showed potential as candidate reference genes in some studies [31, 33]. Hsp70 showed no significant difference by comparing Zn-treated group, Cd-treated group and the CK group (Fig. 4), indicating that Hsp70 might not closely relate to the dose of heavy metals in this study. Moreover, no significant difference was detected by normalization with these three reference genes (Fig. 4). This indicated the suggested reference genes were relatively reliable. Moreover, TBP, the most unstable gene by Refinder, was also used for normalization (Fig. 4). The Hsp70 expression of Cd-treated group was significantly different, lower normalized by TBP than the other three stable ones. This indicated that improper reference gene might lead to problematic results.

## Discussion

Here, we identified the appropriate reference genes for gene expression analysis in *C. megacephala* under five experimental conditions with nine candidate reference genes (Actin, Rpl8, GAPDH, EF1, α-TUB, β-TUB, TBP, Rps7 and 18S). To our knowledge, this is the first study to evaluate different candidate reference genes for gene expression analysis under various experimental conditions in *C. megacephala*. Until now, only a few studies were on the reference evaluation in blowfly species. Cardoso et al. 2014 suggested that Actin, GAPDH, and Rpl49 were the most stable genes among life stages in three Calliphoridae species (*Cochliomyia hominivorax* Coquerel, *C. macellaria* Fabricius, and *C. albiceps* Wiedemann) by designing primers with conserved regions of six candidate reference genes [34]. In the Australian sheep blowfly, *Lucilia cuprina*, 18S, 28SrRNA, GST1 (Glutathione-S-transferase), β-TUB and RPLPO (Acidic ribosomal phosphoprotein PO) were suggested to be an ideal set of genes for data normalization across all life stages, while GAPDH was proven to be a poor reference gene [35]. Our outcomes would provide valuable information for gene expression studies of this synanthropic fly.

It is necessary to validate the feasibility of the selected reference genes in this study. Hsp70 was used as target gene by normalization with the selected α-TUB, β-TUB and GAPDH. It is known that heat shock proteins are stress-induced and participate in various biological progresses. We speculated that Hsp70 might be induced by Zn and Cd. However, no significant difference in Hsp70 expression was found between the heavy metal groups and untreated controls by normalization by any of the selected reference genes. This may indicate that the slight dose of Zn and Cd might not influence the expression of Hsp70. The normalization results of the selected reference genes showed no significant differences. Additionally, Hsp70 might be a potential reference gene in some experimental conditions. The results of this study have been used in gene expressions in the development and tissues of *C. megacephala* [21].

No ideal reference genes were stable enough for the entire subset of developmental stages. Moreover, the ranking orders fluctuate according to different software. For example, Rps7 was calculated to be top (1st) by Normfinder while top 3 by Bestkeeper. Firstly, for all the four methods used in the study, they have their own formula and algorithms. Many reference evaluation papers have discussed this thoroughly [30, 31]. It is possible to achieve diverse results accordingly. For developmental stages, metamorphosis happens violently in insects, which might be an obstacle for seeking stable housekeeping genes. For example, candidate reference genes showed relatively unstable expression with development in *Nilaparvata lugens*, *Solenopsis invicta* and *Tribolium castaneum* [36–38]. Some research found a relatively stable reference gene for developmental stages [39]. In any case, stable reference genes of *C. megacephala* need more experiments to find suitable internal control genes for gene expression studies in related studies. According to this study, ideal reference genes were suggested. However, reference genes expression was more stable in experiment subset of different food feeding, drugs-treated, heavy metals-treated groups and different tissues than developmental stages. It might be possible because these subsets of different samples were at least in the same insect stage.

We would like to discuss more about the possible applications of this insect than the stability of a certain reference gene of it. Rather, it is not because the latter point is not so important or necessary. In fact, it is quite practical for reference values for relevant researchers. It is because the former point would raise concern on this insect and encourage more researchers. Firstly, some of the experiments were aimed to cover the basic aspects of *C. megacephala*, such as developmental studies, food resources and tissues. Developmental studies of *C. megacephala* have long been the most written topics for forensic use. *C. megacephala* could develop on several types of meat under laboratory conditions [40]. Different food for larvae would affect the development period as introduced before. Moreover, tissue expression of target gene is an essential part of regular gene functional research. The experiments of development, food resources and tissues would promote the research levels from biology to molecular biology. Secondly, experiments with artificial diets were designed to consider the possible exposure to drugs and heavy metals for *C. megacephala*. Drugs in a corpse would influence *C. megacephala* in the natural world and further affect the accuracy of PMI. For example, diazepam altered the development of *Chrysomya* [41], and human contraceptive in adult *C. megacephala* has been confirmed to cause ovariole reduction, less matured ovariole and affected cellular changes in testes and ovariole of the offspring [42]. The experiments of drugs in this paper would help with the basic molecular biology researches in drug toxicology and further play a role in accurate estimation of PMI. In the ecosystem, *C. megacephala* could naturally breed on different types of filth [2], which now were used to transform organic waste into protein or biodiesel production [43, 44]. During these processes, toxic heavy metals would accumulate [45] and become risk factors that *C. megacephala* have to face. Actually, the organic waste, which was transformed by *C. megacephala*, such as feces from animal husbandry, always contain a certain amount of metals [46]. The safety of *C. megacephala-*organic waste transformed system would raise health concerns. It was expected that the probing into valid reference gene under heavy metal exposure of *C. megacephala* would work a little in related molecular biology studies and provide potential solutions to a clean production system.

## Conclusion

It is fundamental but vital to find stable reference genes for qPCR in *C. megacephala*. This is the first report, to our knowledge, to evaluate candidate reference genes in *C. megacephala*. Based on Refinder, EF1 was the most suitable reference gene of developmental stages, food and tissues. Rps7, 18S and GAPDH were the most suitable reference genes of drugs and heavy metals, respectively. According to geNorm and the moneywise discipline, the suitable reference gene pairs were recommended as below: developmental stages (EF1, Rps7 and α-TUB); food resource (Actin, GAPDH and EF1); drugs treatment (Rpl8 and EF1); heavy metals treatment (α-TUB and β-TUB); and tissues (EF1 and TBP). Our outcomes would facilitate the gene studies of related biological processes and deepen the understanding in physiology, toxicology, and especially medical and forensic entomology of *C. megacephala*. However, it would be more appropriate for a likely audience to verify these results flexibly than to apply them directly.

## Methods

### Insects

The oriental blowfly, *C. megacephala* was supplied from a laboratory population from Hubei Insect Resources Utilization and Sustainable Pest Management Key Laboratory. The adults were reared in mesh cages and fed on sugar and water. Fresh pork was put into the cages to collect eggs. Eggs together with the pork were taken out and reared until pupation on fresh meat. Larvae were reared with pork except for the diet treatments. Insects were kept for their entire life cycle in 25 ± 2 °C, 60 ± 5 % relative humidity and photoperiod cycle of 13 L:11D.

### Bioassays

#### Developmental stages

Samples were collected from eggs, three larval stages, pupae and adults of *C. megacephala* for stability evaluation of reference genes. Four sample replicates of eggs, 15 first instar larvae, 10 s instar larvae, 5 third instar larvae, 5 pupae and 4 adults (half males and half females) of *C. megacephala* were each collected into 1.5 mL centrifuge tubes and immediately frozen in liquid nitrogen until use. No obvious death was detected during the sample collection.

#### Food resource

Samples were collected from third instar larvae of *C. megacephala* feeding with different kinds of meat for stability evaluation of reference genes. Eggs were collected with lean pork as introduced and then moved to sufficient pork, fish or chicken as larval food sources. When the larvae reached the third instar, 4 sample replicates of 5 larvae were collected and stored as introduced above. No obvious death was detected during the sample collection.

#### Drugs

Samples were collected from third instar larvae of *C. megacephala* exposed under Estazolam (a depressant), Marvelon (a human contraceptive) and normal conditions from egg stage for stability evaluation of reference genes. Estazolam (Huazhong Pharmaceutical Ltd, China) was obtained from health center of Huazhong Agricultural University (HZAU) with essential purchase procedures. Marvelon (N.V. Organon, Netherlands) was obtained from a drugstore. Estazolam and Marvelon were powdered and added in the form of a suspension in water to an artificial diet of *C. megacephala* with a dose of 1 mg drug/150 mL feedstuff (Recipe listed in the Additional file 4). One hundred eggs were counted and introduced to a glass bottle containing 150 mL of artificial feed stuff. For each treatment group, four sample replicates of 5 individuals were collected when larvae reached the third instar. All the samples were handled and stored as introduced above. No obvious death was detected between the treated and control groups.

#### Heavy metal

Samples were collected from third instar larvae of *C. megacephala* exposed under cadmium (Cd^2+^), zinc (Zn^2+^) and normal conditions from egg stage for stability evaluation of reference genes. Heavy metals were added to the larval medium in forms of CdCl_2_ and ZnCl_2_ suspended in water. Mother solutions of CdCl_2_ and ZnCl_2_ were prepared in the concentrations of 1 mM/L. Twenty microliters of the mother solutions were diluted with water and added into the 150 mL of the artificial diet. Then 100 eggs were introduced into feedstuff as introduced above. For each treatment group, 4 sample replicates of 5 individuals were collected when larvae reached the third instar. All the samples were handled and stored as introduced above. No obvious death of was detected between the treated and control groups.

#### Tissues

Samples were collected from adult tissues of *C. megacephala* for stability evaluation of reference genes. Head, thorax, abdomen, legs and wings were obtained from both sexes with a pair of tweezers. For each tissue, 4 sample replicates of 20 insects (half males and half females) each were collected. All the samples were handled and stored as introduced above.

### Total RNA isolation and cDNA synthesis

Total RNA was prepared using TRIzol® Reagent (Ambion®, Life technologies, U.S.). Nanodrop2000 (Thermo Scientific, U.S.) was used to check the concentration and quality of each RNA sample. Qualified RNAs (OD260/280: 1.9 to 2.1) were put into further cDNA synthesis with First Strand cDNA Synthesis Kit (NEWBIOTech., Canada). cDNAs was stored in the −20 °C refrigerator before use.

### Reference gene selection and primer design

The involved reference genes were downloaded from the NCBI (http://www.ncbi.nlm.nih.gov/) and transcriptome data of *C. megacephala*. The candidate primers were designed by online primer design tool for Real-time PCR (http://www.idtdna.com/site) with default settings. The Primers were synthesized by Newtsingke Biotech (Wuhan, China). The length and identity of PCR products were assessed with gel electrophoresis and sequence analysis. Then sensitivity, specificity, and capacity of the right primers were testified by melting curve and standard curve with the Bio-Rad iQ5 Optical System. Then the remaining qualified primers were used for further qPCR evaluation. A standard curve was achieved for each gene by five-fold serial dilution of the templates. The qualified primers used for qPCR, their PCR efficiency and regression coefficient and were shown in Table 1.

### qPCR

cDNAs from each treatment were used for qPCR using Real Master Mix (SYBR™ Green I) (NEWBIOTech., Canada). Amplification was performed in a 20 μl volume with 2 μl of cDNA and 100 nM of each primer. Polymerase Chain Reactions were set as follows: initial denaturation temperature, held at 95 °C for 30 s, followed by 40 cycles at 95 °C for 5 s and 58/59 °C for 30 s, and 72 °C for 2 min to terminate the reaction. Finally, a melting curve analysis was also applied to confirm the consistency and specificity of the amplicon from 55 °C to 95 °C. Three biological replicates were done for individual treatment. Initially, 10 candidate reference genes were investigated.

### Gene expression stability analysis

All treatments were performed in three biological replicates. Stability of the nine candidate reference genes were comprehensively evaluated using RefFinder (http://www.leonxie.com/referencegene.php) with average Ct value. RefFinder is a free software online that combines the currently available major computational programs (geNorm, Normfinder, BestKeeper, and the ΔCt method) and give comprehensive ranking orders based on them [26–29]. The ranking orders were weighed with the geometric mean; and candidate genes with a lower geometric mean were thought to be more stable. geNorm was also used to give suggestions on the number of reference genes. Quantities were transformed into a linear scale and the highest relative quantity for each gene was set to one, which was used as input data for geNorm. Firstly, M value (the expression stability value) was calculated for every gene and then V value (the pairwise variation) was calculated by comparing values with each other. Candidate reference genes are given an order by o M values [47]. Finally, geNorm suggests a minimum of number of reference genes by V value for normalization. If V_n/n+1_ is less than 0.15, no more reference genes are needed for normalization [26].

### Reference gene validation

Hsp70 (AGL51120) was selected as target gene for stability validation: F-ATGTCTAAAGCTCCTGCTATTGGT, R- TTAATCGACTTCTTCGATGGTGG). The relative expression level of Hsp70 was normalized with 3 top identified stable reference genes and the worst one by Refinder under heavy metal exposureby 2^-ΔΔCT^ method. Ducan’s multiple range test was used to measure the significance.

## Additional files

Additional file 1: Table S1. Recipe of the artificial feedstuff of *C. megacephala* larvae (DOCX 18 kb) Additional file 2: Table S2. Ranking orders of the candidate reference genes of *C. megacephala* within all larval samples. Ct values within all larval samples were combined together, and ranking orders of the candidate reference genes were calculated by RefFinder. (DOCX 16 kb) Additional file 3: Table S3. Ranking orders of the candidate reference genes of *C. megacephala* within all pupal samples. Ct values within all pupal samples were combined together, and ranking orders of the candidate reference genes were calculated by RefFinder. (DOCX 17 kb) Additional file 4: Table S4. Ranking orders of the candidate reference genes of *C. megacephala* within all adult samples. Ct values within all adult samples were combined together, and ranking orders of the candidate reference genes were calculated by RefFinder. (DOCX 17 kb)
