# Supplementary material for: Reference gene stability of a synanthropic fly, Chrysomya megacephala
Source: Parasit Vectors. 2015 Oct 29;8:565. doi: 10.1186/s13071-015-1175-9 (PMC4625446; doi:10.1186/s13071-015-1175-9)
Supplement: Additional file 1: Table S1. — Recipe of the artificial feedstuff of C. megacephala larvae (DOCX 18 kb) [file 13071_2015_1175_MOESM1_ESM.docx]

**Table S1 Ranking orders of** **the candidate reference genes of *C. megacephala* within all larvae samples**

| **Rank** | **RefFinder** | **ΔCt** | **Bestkeeper** | **NormFinder** | **geNorm** |
| --- | --- | --- | --- | --- | --- |
| **1** | Rps7 | Rps7 | Rps7 | β-TUB | β-TUB\|Rps7 |
| **2** | β-TUB | TBP | β-TUB | Rps7 |  |
| **3** | TBP | β-TUB | TBP | TBP | TBP |
| **4** | 18S | 18S | 18S | 18S | 18S |
| **5** | EF1 | EF1 | EF1 | EF1 | EF1 |
| **6** | GAPDH | GAPDH | α-TUB | α-TUB | GAPDH |
| **7** | α-TUB | Rpl8 | GAPDH | GAPDH | Rpl8 |
| **8** | Rpl8 | α-TUB | Rpl8 | Rpl8 | α-TUB |
| **9** | Actin | Actin | Actin | Actin | Actin |
