# Supplementary material for: Reference gene stability of a synanthropic fly, Chrysomya megacephala
Source: Parasit Vectors. 2015 Oct 29;8:565. doi: 10.1186/s13071-015-1175-9 (PMC4625446; doi:10.1186/s13071-015-1175-9)
Supplement: Additional file 2: Table S2. — Ranking orders of the candidate reference genes of C. megacephala within all larval samples. Ct values within all larval samples were combined together, and ranking orders of the candidate reference genes were calculated by RefFinder. (DOCX 16 kb) [file 13071_2015_1175_MOESM2_ESM.docx]

**Table S2 Ranking orders of** **the candidate reference genes of *C. megacephala* within all pupal samples**

| **Rank** | **RefFinder** | **ΔCt** | **Bestkeeper** | **NormFinder** | **geNorm** |
| --- | --- | --- | --- | --- | --- |
| **1** | Rpl8 | EF1 | 18S | Rpl8 | Actin\|GAPDH |
| **2** | EF1 | Rpl8 | β-TUB | α-TUB |  |
| **3** | α-TUB | α-TUB | Rpl8 | Rps7 | EF1 |
| **4** | Rps7 | Rps7 | TBP | EF1 | Rpl8 |
| **5** | Actin | β-TUB | α-TUB | β-TUB | Rps7 |
| **6** | β-TUB | TBP | Rps7 | TBP | α-TUB |
| **7** | GAPDH | Actin | EF1 | Actin | TBP |
| **8** | 18S | GAPDH | Actin | GAPDH | β-TUB |
| **9** | TBP | 18S | GAPDH | 18S | 18S |
