# Supplementary material for: Reference gene stability of a synanthropic fly, Chrysomya megacephala
Source: Parasit Vectors. 2015 Oct 29;8:565. doi: 10.1186/s13071-015-1175-9 (PMC4625446; doi:10.1186/s13071-015-1175-9)
Supplement: Additional file 3: Table S3. — Ranking orders of the candidate reference genes of C. megacephala within all pupal samples. Ct values within all pupal samples were combined together, and ranking orders of the candidate reference genes were calculated by RefFinder. (DOCX 17 kb) [file 13071_2015_1175_MOESM3_ESM.docx]

**Table S3 Ranking orders of** **the candidate reference genes of *C. megacephala* within all adult samples**

| **Rank** | **RefFinder** | **ΔCt method** | **Bestkeeper** | **NormFinder** | **geNorm** |
| --- | --- | --- | --- | --- | --- |
| **1** | EF1 | EF1 | Rps7 | EF1 | EF1\|TBP |
| **2** | Rpl8 | Rpl8 | Actin | Rpl8 |  |
| **3** | TBP | TBP | α-TUB | TBP | Rpl8 |
| **4** | β-TUB | GAPDH | β-TUB | GAPDH | GAPDH |
| **5** | Rps7 | β-TUB | EF1 | β-TUB | β-TUB |
| **6** | GAPDH | α-TUB | TBP | α-TUB | α-TUB |
| **7** | α-TUB | Actin | Rpl8 | Actin | Actin |
| **8** | Actin | Rps7 | GAPDH | Rps7 | Rps7 |
| **9** | 18S | 18S | 18S | 18S | 18S |
