# Supplementary material for: Reference gene stability of a synanthropic fly, Chrysomya megacephala
Source: Parasit Vectors. 2015 Oct 29;8:565. doi: 10.1186/s13071-015-1175-9 (PMC4625446; doi:10.1186/s13071-015-1175-9)
Supplement: Additional file 4: Table S4. — Ranking orders of the candidate reference genes of C. megacephala within all adult samples. Ct values within all adult samples were combined together, and ranking orders of the candidate reference genes were calculated by RefFinder. (DOCX 17 kb) [file 13071_2015_1175_MOESM4_ESM.docx]

**Table S1 Recipe of the artificial feedstuff of larval** ***C. megacephala***

| **Ingredient** | **Amount** |
| --- | --- |
| Wheat bran | 600 g |
| Fish powder | 100 g |
| Milk powder | 40 g |
| Yeast extract | 8 g |
| Tryptone | 15 g |
| ddH_2_O | 1500 mL |
| Mix well, sub-package and sterilize by autoclaving, immediate use or store at 4 °C | |
